# Multiple Photolyases Protect the Marine Cyanobacterium *Synechococcus* from Ultraviolet Radiation

**DOI:** 10.1128/mbio.01511-22

**Published:** 2022-07-20

**Authors:** Allissa M. Haney, Joseph E. Sanfilippo, Laurence Garczarek, Frédéric Partensky, David M. Kehoe

**Affiliations:** a Department of Biology, Indiana University, Bloomington, Indiana, USA; b Sorbonne Université, CNRS, Station Biologique de Roscoff (SBR), UMR 7144 Adaptation and Diversity in the Marine Environment, Roscoff, France; University of Hawaii at Manoa

**Keywords:** DNA photolyase, *Synechococcus*, UV light, cyanobacteria, marine microbiology

## Abstract

Marine cyanobacteria depend on light for photosynthesis, restricting their growth to the photic zone. The upper part of this layer is exposed to strong UV radiation (UVR), a DNA mutagen that can harm these microorganisms. To thrive in UVR-rich waters, marine cyanobacteria employ photoprotection strategies that are still not well defined. Among these are photolyases, light-activated enzymes that repair DNA dimers generated by UVR. Our analysis of genomes of 81 strains of *Synechococcus*, *Cyanobium*, and *Prochlorococcus* isolated from the world’s oceans shows that they possess up to five genes encoding different members of the photolyase/cryptochrome family, including a photolyase with a novel domain arrangement encoded by either one or two separate genes. We disrupted the putative photolyase-encoding genes in *Synechococcus* sp. strain RS9916 and discovered that each gene contributes to the overall capacity of this organism to survive UVR. Additionally, each conferred increased survival after UVR exposure when transformed into Escherichia coli lacking its photolyase and SOS response. Our results provide the first evidence that this large set of photolyases endows *Synechococcus* with UVR resistance that is far superior to that of E. coli, but that, unlike for E. coli, these photolyases provide *Synechococcus* with the vast majority of its UVR tolerance.

## INTRODUCTION

The penetration of photosynthetically active radiation (400 to 700 nm) into the upper layer of the oceans is essential to sustain phytoplankton photosynthetic activity, which accounts for approximately half of the Earth’s global primary productivity (1). However, in near-surface waters, excessive amounts of photosynthetically active radiation as well as UV radiation (UVR) negatively affect photosynthesis and cell viability (2–6). UVR has been shown to have deleterious impacts on cellular structures and metabolic processes of phytoplanktonic cells in culture (7–10), and several studies have also identified damages caused to DNA by such radiation in natural populations of marine phytoplankton (11–13).

Marine photosynthetic organisms vary dramatically in their ability to survive UVR exposure. The picocyanobacterium *Synechococcus* is among the most capable of tolerating and recovering from the damaging effects of UVR (14). As the second most abundant phytoplanktonic organism after *Prochlorococcus*, with an estimated global abundance of 7 × 10^26^ cells and a contribution to global net marine primary productivity as high as 16% (15, 16), marine *Synechococcus* are clearly major contributors to the carbon cycle and the marine food web. This group is characterized by compact genomes, which typically range in size from approximately 2.1 to 3.3 Mbp (17).

A variety of different mechanisms have evolved to repair UVR-damaged DNA (18). These repair processes are divided into two main classes: those which do not require light (light-independent repair, or LIR), such as nucleotide excision repair and base excision repair, and those that require light during or immediately after UVR exposure, a process called photoreactivation (19), which is carried out by enzymes called DNA photolyases (20–23). The extent to which LIR versus photoreactivation processes are used for DNA repair varies from species to species (20, 24).

Members of the photolyase/cryptochrome family have a variety of functions and are found in both prokaryotes and eukaryotes. There are two groups of DNA photolyases. Both use blue light as the energy source to catalyze the reaction. One group, called CPD photolyases, repairs cyclobutane pyrimidine dimers (CPDs), and the other group, called (6-4) photolyases, repairs pyrimidine-pyrimidone (6-4) photoproducts (19, 25). Many cryptochromes also absorb blue light and have light sensing roles, while others are not photoreceptors and operate in circadian systems and magnetoperception (26–28). DNA photolyase function and structure have been intensively studied, particularly in the enteric bacterium Escherichia coli, which is arguably the best-understood model system for how DNA is repaired in response to UVR damage. Photoreactivation in E. coli is carried out by a single type of photolyase, which is encoded by the *phr* gene (29, 30). The initial determination of the crystal structure of the *E. coli* photolyase (31, 32) showed that it is composed of two domains. One, generally located in the C-terminal part of the protein, binds to the DNA lesion and in all known cases contains flavin adenine dinucleotide (FAD), which acts both as a cofactor and as the primary chromophore (33). The second, less conserved, N-terminal domain contains an additional chromophore, which acts as a light harvesting antenna, providing additional blue light energy to drive the reaction. All subsequently examined photolyases have also been found to consist of these two domains, although a variety of chromophores has been found to be associated with the second domain in different members of the photolyase/cryptochrome family. Thus far, these have been found to be a derivative of either a flavin such as 5-deazaflavin in 8-hydroxy-7,8-didemethyl-5-deazariboflavin (8-HDF), a folate such as methenyltetrahydrofolate (MTHF), or lumazine, such as 6,7-dimethyl-8-ribityllumazine (DMRL) (34).

In the present study, we examined the genome of the marine *Synechococcus* sp. strain RS9916 (here called 9916), which was isolated from the Gulf of Aqaba in the Red Sea (35) and is a model organism for light color acclimation studies (36–39). Despite its relatively small size, this genome contains five genes that encode complete or partial proteins belonging to the cryptochrome/photolyase family (20, 40, 41). Our identification of five genes in 9916 encoding possible photolyase or cryptochrome proteins raised questions about the UVR tolerance of this specific strain, the functions of these proteins, and the extent to which these genes are also found in other strains of marine picocyanobacteria (unicellular cyanobacteria smaller than 2 to 3 μm). Here, using comparative genomics, we determined that these genes are highly conserved in the genomes of all marine *Synechococcus* isolates sequenced to date, and some are also found in *Prochlorococcus* and *Cyanobium* genomes. Our physiological studies demonstrate that 9916 is far more UVR tolerant than E. coli, but that unlike E. coli, which relies heavily on LIR processes, its recovery from damages caused by UVR predominantly occurs via photoreactivation-mediated processes. Using molecular genetic approaches, we show that each of these genes confers 9916 with a significant ability to survive UVR, and when expressed in E. coli, confers those cells with additional protection from UVR damage, in some cases in a light-dependent fashion. Our results demonstrate that marine *Synechococcus* strains are well adapted to survive the strong UVR exposure they experience in the surface layer of the oceans and use multiple putative photolyases to achieve this.

## RESULTS

### Diversity and phylogeny of members of the photolyase/cryptochrome family in marine and brackish picocyanobacteria.

**FIG 1:**
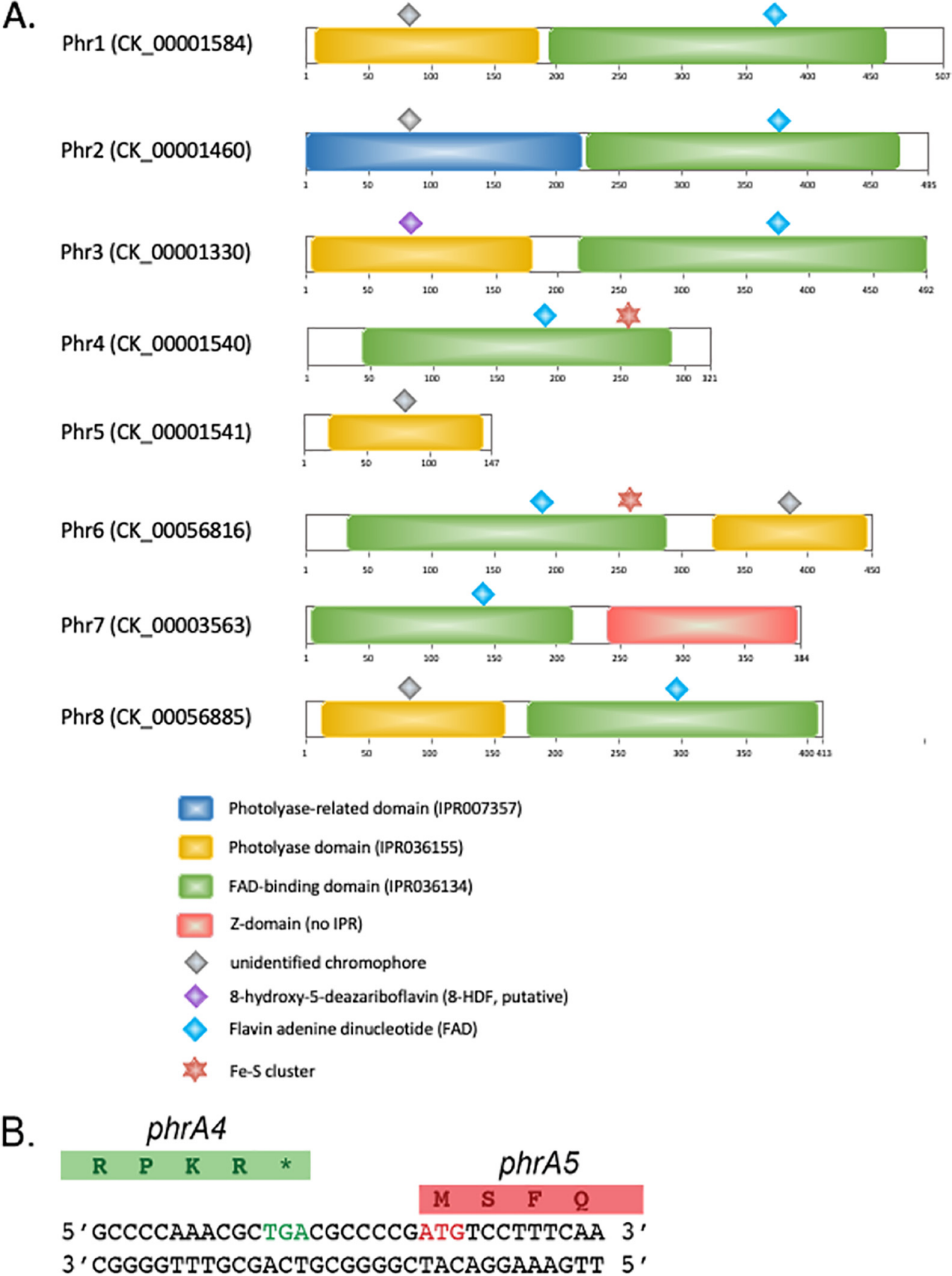
Structural domains and bound chromophores for the eight members of the cryptochrome/photolyase family found in marine and brackish picocyanobacteria and illustration of the *phr4/phr5* junction. (A) Diagrams show the positions of the different protein domains, as predicted from InterProScan (108) in representative sequences of the eight Cyanorak v2.1 clusters of likely orthologous genes (CLOGs), indicated between brackets after the protein names. Sequences shown here are from *Synechococcus* sp. RS9916 for Phr1-5, from *Cyanobium* sp. NS01 for Phr6, from *Prochlorococcus* sp. MIT9302 for Phr7, and from *C. gracile* PCC 6307 for Phr8. By analogy with the freshwater *Synechococcus* sp. (formerly Anacystis nidulans) strain PCC 6301 (109), we assume that an 8-hydroxy-5-deazaflavin (8-HDF), represented as a purple diamond, is bound to the DNA photolyase domain (InterPro accession no. IPR036155; 109) of Phr3. The chromophores bound to the other DNA photolyase domains shown, and to the photolyase PhrB-like domain (IPR007357), have not yet been identified and are denoted by a gray diamond. Additionally, blue diamonds indicate that the amino acids involved in flavin binding to the FAD binding domain (IPR036134), identified in *Synechococcus* sp. PCC 6301 Syc1392_c (Y228, T240, S241, L243, S244, W280, R287, T346, N349, D380, D382, A385, and N386), are conserved in the FAD domains of all CLOG members (e.g., Y247, T259, S260, L262, S263, W299, R306, W365, N368, D399, D401, A404, and N405 in 9916 Phr3). Finally, red stars indicate that the corresponding FAD domains contain the residues necessary to bind an Fe-S complex (e.g., in RS9916 Phr4: C169, C254, C257, and C263), as found in the (6-4) photolyase from A. tumefaciens (46). (B) DNA and translated protein sequences from the genomic region spanning *phr4* and *phr5* in 9916. The DNA sequence encoding the stop codon (asterisk) of *phr4* is in green, while the start codon of *phr5* is in red.

**FIG 2:**
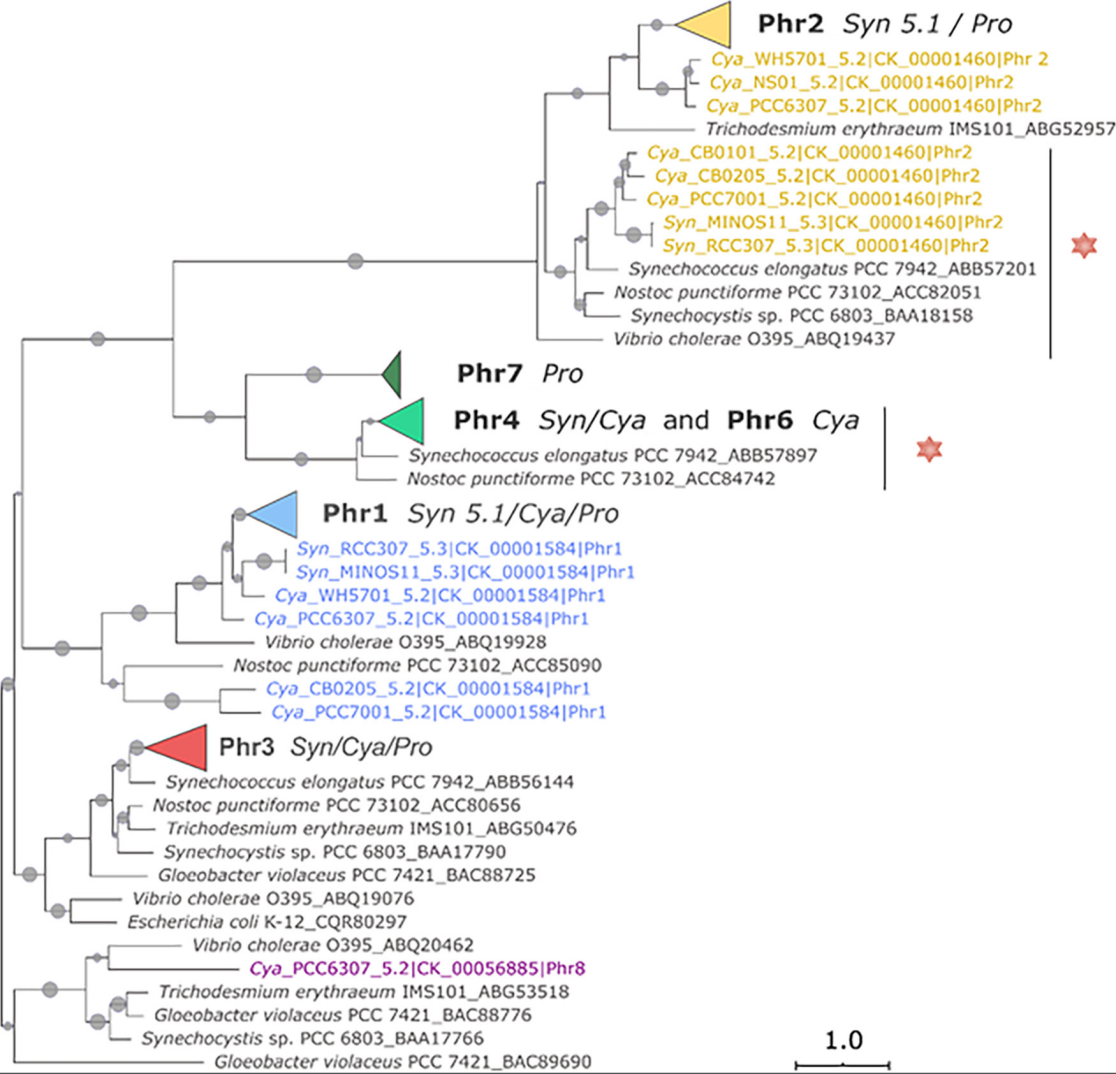
Maximum likelihood phylogenetic tree of the cryptochrome/photolyase family based on the FAD domain. Marine picocyanobacteria sequence members retrieved from 81 nonredundant genomes from Cyanorak v2.1 (16) are shown in colors, with monophyletic groups within each protein member being collapsed as colored triangles (the noncollapsed tree is shown in Fig. S1). Outgroup sequences are shown in black. Picocyanobacterial sequence names include the abbreviation of the genus (Pro, *Prochlorococcus*; Syn, *Synechococcus*; Cya, *Cyanobium*), strain name, and subcluster *sensu* Herdman et al. (110), as well as the Cyanorak CLOG number and the proposed protein designation as in Fig. 1 and Table S1. Plain gray circles on branches of the tree correspond to maximum likelihood bootstrap values ranging from 70 to 100% (lower values have been omitted). The red star indicates the members that possess the four conserved cysteine residues necessary to bind an Fe-S cluster, as found, for example, in the (6-4) photolyase from A. tumefaciens (46) and V. cholerae O395 (Table S2). Note that Phr5 is not shown since it does not possess a FAD domain.

Analysis of the putative proteins encoded within the 81 nonredundant genomes of *Prochlorococcus*, *Synechococcus*, and *Cyanobium* of the Cyanorak v2.1 database, which are representative of a wide variety of marine and brackish habitats (16, 42), revealed the presence of eight different members of the photolyase/cryptochrome family with distinct phyletic patterns, i.e., patterns of presence/absence of these members in each strain (see Fig. S1 in the supplemental material). These proteins, which were provisionally designated Phr1 through Phr8, were analyzed with regard to their domain content and phylogenetic relatedness (Fig. 1 and 2 and Tables S1 and S2).

10.1128/mbio.01511-22.1 FIG S1 Noncollapsed maximum likelihood phylogenetic tree of the cryptochrome/photolyase family based on the FAD domain. Same tree as in Fig. 1 but branches corresponding to marine picocyanobacteria sequence members, retrieved from 81 nonredundant genomes from Cyanorak v2.1 (H. Doré, G. K. Farrant, U. Guyet, J. Haguait, et al., Front Microbiol, 2020, https://doi.org/10.3389/fmicb.2020.567431) are not collapsed. Picocyanobacterial sequence names include abbreviation of the genus (Pro, *Prochlorococcus*; Syn, *Synechococcus*; Cya, *Cyanobium*), strain name, finest taxonomical level for each strain *sensu* (G. K. Farrant, H. Doré, F. M. Cornejo-Castillo, F. Partensky, et al., Proc Natl Acad Sci USA 113:E3365–E3374, 2016, https://doi.org/10.1093/nar/gkaa1147), i.e., subcluster (e.g., 5.2), clade (e.g., VII), or subclade (e.g., IIIa), the pigment type *sensu* (T. Grebert, H. Doré, F. Partensky, G. K. Farrant, et al., Proc Natl Acad Sci USA 115:E2010–E2019, 2018, https://doi.org/10.1073/pnas.1717069115), as well as the Cyanorak CLOG number and the proposed protein designations as in Fig. 1 and Table S1. Series of three numbers at the nodes of the tree correspond to bootstrap values for maximum likelihood, neighbor joining, and maximum parsimony, respectively. For readability, bootstrap values at the extremity of branches are not shown. Red stars indicate that FAD domains of the corresponding members possess the four conserved cysteine residues necessary to bind an Fe-S cluster, for example, as found in the (6-4) photolyase from A. tumefaciens (F. Zhang, P. Scheerer, I. Oberpichler, T. Lamparter, et al., Proc Natl Acad Sci USA 110:7217–7222, 2013, https://doi.org/10.1073/pnas.1302377110) and V. cholerae O395 (Table S2). Download FIG S1, PPT file, 0.7 MB. Copyright © 2022 Haney et al. 2022 Haney et al. https://creativecommons.org/licenses/by/4.0/ This content is distributed under the terms of the Creative Commons Attribution 4.0 International license.

10.1128/mbio.01511-22.8 TABLE S1 Characteristics of strains and genomes used in Fig. 1 and presence/absence of the different putative photolyase genes. Colored squares above gene names refer to the protein domains shown in Fig. 1. Codes between brackets below gene names correspond to the Cyanorak v2.1 cluster number, while the name of the subfamily used in Fig. 1A from H. J. Emmerich, M. Saft, L. Schneider, D. Kock, et al. Nucleic Acids Res 48:12845–12857, 2020, https://doi.org/10.1093/nar/gkaa1147, and other classically used names (between brackets) are indicated below. RCC, Roscoff Culture Collection. Download Table S1, XLS file, 0.1 MB. Copyright © 2022 Haney et al. 2022 Haney et al. https://creativecommons.org/licenses/by/4.0/ This content is distributed under the terms of the Creative Commons Attribution 4.0 International license.

10.1128/mbio.01511-22.9 TABLE S2 Characteristics of members of the DNA photolyase/cryptochrome family from outgroup reference organisms not included in Cyanorak v2.1. Interpro (IPR) domains (also shown as colored squares, as in Fig. 1), presence of Fe-S cluster, the most related picocyanobacterial gene by phylogeny (Fig. 2) or BlastN, and the most closely related cluster of likely orthologous genes (CLOG) in Cyanorak V2.1 are indicated for each sequence. Column N indicates the name of the subfamily used in Fig. 1A from H. J. Emmerich, M. Saft, L. Schneider, D. Kock, et al. Nucleic Acids Res 48:12845–12857, 2020, https://doi.org/10.1093/nar/gkaa1147, and other classically used names (between brackets). Download Table S2, XLS file, 0.1 MB. Copyright © 2022 Haney et al. 2022 Haney et al. https://creativecommons.org/licenses/by/4.0/ This content is distributed under the terms of the Creative Commons Attribution 4.0 International license.

All marine *Synechococcus* and *Cyanobium* strains were found to contain three open reading frames (ORFs) (*phr1* to *phr3*) that appear to encode full-length proteins. All three proteins appear to possess a C-terminal FAD domain (InterPro accession no. IPR036134). However, while the N-terminal domain of Phr1 and Phr3 apparently is a cryptochrome/DNA photolyase domain (IPR036155) typical of the photolyases found in E. coli or Gloeobacter violaceus (Table S2), the N-terminal domain appears to be a PhrB-like photolyase domain in Phr2 (IPR007357; Fig. 1A and Table S2). Phr2 corresponds to the previously described *Prochlorococcus* (6-4) photolyase called PromaPL, which lacks an iron-sulfur (Fe-S) cluster (43) (Table S1). In addition, all marine *Synechococcus* but only half of the six *Cyanobium* strains possess two additional, adjacent ORFs, *phr4* and *phr5*, which together encode the two domains found in typical photolyases. In all of these strains, these two ORFs are separated by seven G-C base pairs, resulting in a reading frameshift (Fig. 1B). It is not yet clear whether they are cotranslated as a single protein as a result of ribosomal frameshifting, which is well documented in prokaryotes (44), or if two separate polypeptides are produced from these two ORFs. However, if a single protein is produced, the FAD-binding domain would be at the N-terminal end and the DNA photolyase domain at the C-terminal end, which is in the reverse domain order relative to all previously described photolyases (Fig. 1A and Table S1). Interestingly, two *Cyanobium* strains (NS01 and PCC 7001) possess an ORF, designated *phr6*, whose sequence closely matches that resulting from the merging of *phr4* and *phr5* (Fig. 2 and Fig. S1). This provides support for the hypothesis that this reverse domain structure photolyase is functional (Fig. 1A and Table S1). It is also worth noting that Phr2 sequences form two distinct phylogenetic subclades (Fig. 2), but only one of these possesses the four conserved cysteine residues needed to bind an Fe-S cluster that are characteristic of the (6-4) photolyases in Vibrio cholerae and Agrobacterium tumefaciens (45, 46). These conserved cysteines are also found in all Phr4 and Phr6 sequences. In contrast, Phr1, Phr3, and most Phr2 do not possess these residues. Finally, another *Cyanobium*, the freshwater strain C. gracile PCC 6307, displayed a different gene content, consisting of *phr1*, *phr2*, *phr3*, and an additional, more typical member of the photolyase/cryptochrome family, which we call *phr8* (Fig. 1A and Table S1).

For *Prochlorococcus*, three main gene distribution patterns were identified within the 81 genomes listed in Table S1. The genomes of most high-light (HL)-adapted *Prochlorococcus* strains and of all strains within the low-light (LL)-adapted clade LLI, which occur at intermediate depths (47), contain four putative photolyase-encoding ORFs, orthologs of *phr1*, *phr2*, and *phr3* as well as an additional ORF that is not present in marine *Synechococcus/Cyanobium* and that we have designated *phr7*. Phr7 is formed by an N-terminal FAD domain and a C-terminal Z-domain and was recently shown to encode a novel CPD photolyase that acts on single-stranded DNA in Dinoroseobacter shibae and Methylobacterium mesophilicum (48) (Fig. 1). Phylogenetically, Phr7 forms a cluster that is most closely related to Phr4 and Phr6 from *Synechococcus* and *Cyanobium*, although Phr7 sequences do not possess the cysteine residues needed to bind the Fe-S cluster (Fig. 2). Two of the three HLI strains (MED4 and EQPAC1) completely lack *phr1*, while the HLII strain MIT9123 contains a frameshift mutation in both *phr1* and *phr2* (Table S1). The genomes of members of *Prochlorococcus* clades LLII to LLV, which are known to be strictly low-light-adapted and therefore restricted to the base of the euphotic layer (47, 49), completely lack genes encoding members of the cryptochrome/photolyase family, as previously reported based on the analysis of a more limited number of genomes (50).

Finally, examination of the genomic context of *phr1* to *phr5* genes in the 9916 genome shows that while *phr1* is not located near any genes encoding DNA repair proteins, *phr2* is located downstream of an ORF that is predicted to encode UvrC, the endonuclease subunit of the excinuclease UvrABC complex, and *phr3* is located several genes upstream of *phr4* and *phr5* (Fig. S2).

10.1128/mbio.01511-22.2 FIG S2 Genomic context of the five putative photolyase genes of *Synechococcus* RS9916. Note that the genomic location of *phr4* and *phr5* is immediately downstream of *phr3* and that the putative photolyase genes have been numbered according to their position in the 9916 genome. Download FIG S2, PPT file, 0.1 MB. Copyright © 2022 Haney et al. 2022 Haney et al. https://creativecommons.org/licenses/by/4.0/ This content is distributed under the terms of the Creative Commons Attribution 4.0 International license.

### *Synechococcus* 9916 versus E. coli survival after UVR treatment.

**FIG 3:**
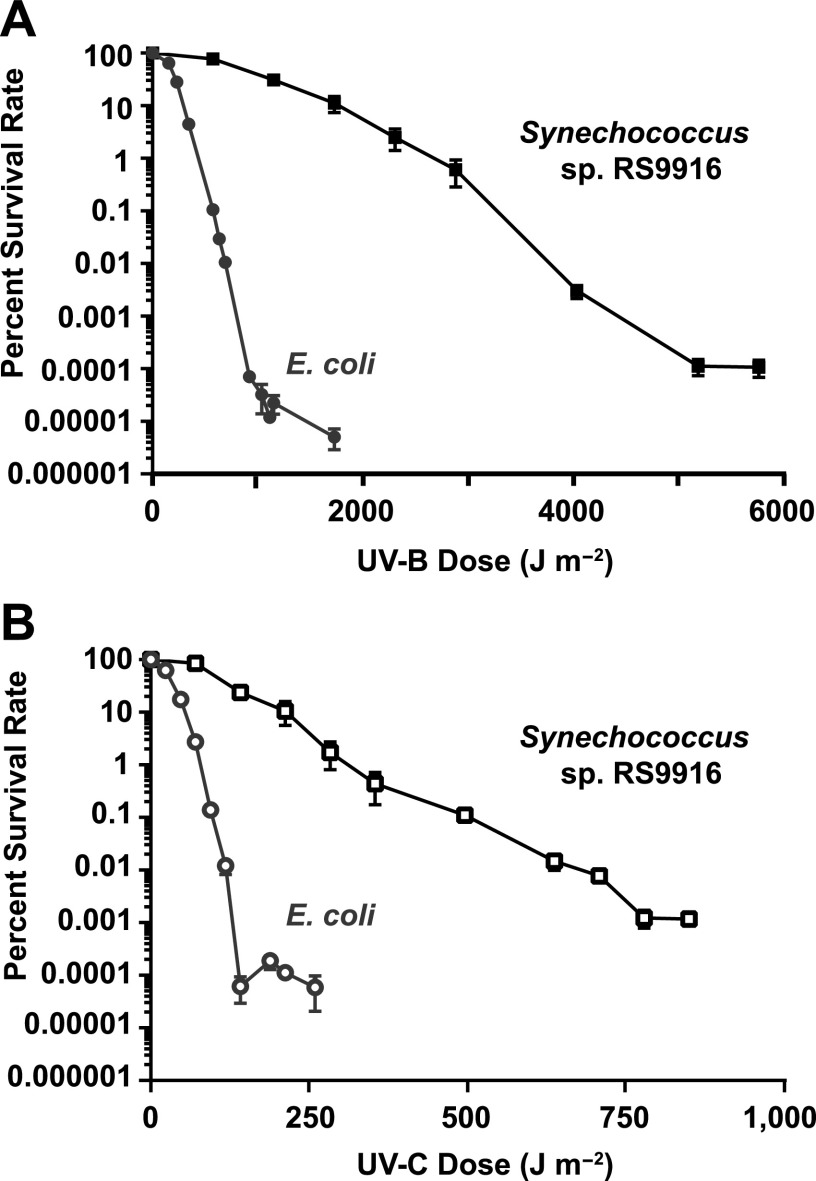
Percent survival rates of two bacterial species after UV-B and UV-C treatment. (A and B) Percent survival rates of E. coli (circles) and marine *Synechococcus* RS9916 (squares) after various doses of (A) UV-B and (B) UV-C radiation followed by continuous white light (WL) exposure. For each species, the number of cells on plates not treated with UV radiation but exposed to continuous WL were used to establish the 100% value. Error bars denote the standard deviation of at least three independent replicates.

Given the presence of the *phr1* to *phr5* genes in all marine *Synechococcus* subcluster 5.1 members (17) (Table S1), we have investigated the role of their products using 9916 as a model organism. We examined the extent to which 9916 survived exposure to a range of UV-B (306 ± 5 nm) and UV-C (254 ± 5 nm) radiation relative to E. coli, which has been extensively studied for its UV radiation response (51–53). E. coli M2 cells, which have normal DNA repair abilities, were used as wild-type cells for this experiment (Table 1). All treatments were immediately followed by continuous exposure to 1 h of white light (WL) to permit photoreactivation (54). E. coli was much more sensitive than 9916 to both UV-B and UV-C radiation, with a million times lower percent survival rate after exposure to 1,000 J m^−2^ of UV-B and a 100,000 times lower percent survival rate after exposure to 250 J m^−2^ of UV-C (Fig. 3A and B). The 1, 000 J m^−2^ of UV-B was delivered over 8.7 min, and the 250 J m^−2^ of UV-C was delivered over 1.8 min. This demonstrates that for the strains examined, marine *Synechococcus* is vastly more resistant to the damaging effects of both UV-B and UV-C radiation than is E. coli when WL is subsequently provided.

**TABLE 1:** Strains, plasmids, and primers used in this study^*a*^

Strain, plasmid, or primer	Description
Strains	
WT RS9916	*Synechococcus* sp. RS9916, isolated from the Red Sea
Control	Kan^r^, mini-Tn5 insertion in uncharacterized gene *RS9916*_*32112*
*phr1*-	Kan^r^, plasmid insertion disrupting *RS9916_27184*
*phr2*-	Kan^r^, plasmid insertion disrupting *RS9916_30014*
*phr3*-	Kan^r^, plasmid insertion disrupting *RS9916_38901*
*phr4*-	Kan^r^, plasmid insertion disrupting *RS9916_38946*
WT *E. coli*	*E. coli* M2 (MG1655)
*lexA3-Δphr*	E. coli SOS null allele of *lexA*, *phr* deleted
E-Cont	Amp^r^, *lexA3*-Δ*phr*, contains pBAD24 plasmid
lexA3+phr1	Amp^r^, *lexA3*-Δ*phr*, contains pBAD24 with *RS9916_27184*
lexA3+phr2	Amp^r^, *lexA3*-Δ*phr*, contains pBAD24 with *RS9916_30014*
lexA3+phr3	Amp^r^, *lexA3*-Δ*phr*, contains pBAD24 with *RS9916_38901*
lexA3+phr4/phr5	Amp^r^, *lexA3*-Δ*phr*, contains pBAD24 with *RS9916_38946/38941*
Plasmids	
pMUT100	Kan^r^ suicide vector used for homologous recombination
pRL528	Chl^r^, helper plasmid, carries *mob*
pRK24	Amp^r^, conjugal plasmid, RK2 derivative
pMUTphr1	pMUT100 derivative, for disruption of *phr1*
pMUTphr2	pMUT100 derivative, for disruption of *phr2*
pMUTphr3	pMUT100 derivative, for disruption of *phr3*
pMUTphr4	pMUT100 derivative, for disruption of *phr4*
pBAD24	Amp^r^, autonomously replicating plasmid used in E. coli
Primers (5′ to 3′)	
NheI-*phr1*-for	GGGGTTGAAACGACGCGAGGGT
SphI-*phr1*-rev	CTCCTTCCAGGCCTGAAACCGCT
BamHI-*phr2*-for	ATAGGATCCTCTGAAAGGACAGGGCTTTGAGGT
BamHI-*phr2*-rev	AACTCCAGAACAAGCCATCCCAGA
BamHI-*phr3*-for	ATAGGATCCTCACCGGTGTGTATGTGCTGGAT
BamHI-*phr3*-rev	ATAGGATCCTGCATCCAACCGGTTTCATTGAGC
NheI-*phr4*-for	GCGGCTAGCTGGATCCGCCATGGAGTGCTCA
SphI-*phr4*-rev	GAGGCATGCCTTTGCTGTACCGCTCCAGGTTG
*Phr1*-test-rev	GAGAAGCTTCTAAAGCTCCAGCTGCAGTTGCTGATC
*Phr2*-test-rev	GAGGAGCTCTCAGTTCAAGCCGTCAAGAAACTGTGATGC
*Phr3*-test-rev	GAGAAGCTTTCAGCTGCGAATCGTGGCGTAAAGCG
*Phr4*-test-rev	GAGAAGCTTTCAGCGTTTGGGGCGGGCGG
Interruption *phr1 F*	GCGGCTAGCGGGGTTGAAACGACGCGAGGGT
Interruption *phr1 R*	GAGGCATGCCTCCTTCCAGGCCTGAAACCGCT
Interruption *phr2 F*	ATAGGATCCTCTGAAAGGACAGGGCTTTGAGGT
Interruption *phr2 R*	AACTCCAGAACAAGCCATCCCAGA
Interruption *phr3 F*	ATAGGATCCTCACCGGTGTGTATGTGCTGGAT
Interruption *phr3 R*	ATAGGATCCTGCATCCAACCGGTTTCATTGAGC
Interruption *phr4 F*	GCGGCTAGCTGGATCCGCCATGGAGTGCTCA
Interruption *phr4 R*	GAGGCATGCCTTTGCTGTACCGCTCCAGGTTG
pMUT100-test-for	ATAGGCTTGGTTATGCCGGTACTGC
pMUT100 test-rev	ACTGGGCTGCTTCCTAATGCAGGAGT
Int-test-rev	ACTCCTGCATTAGGAAGCAGCCCAGT

a WT, wild-type.

### *Synechococcus* 9916 versus E. coli light-dependent survival after UVR treatment.

The existence of both photoreactivation and LIR systems in bacteria, as well as the WL treatment provided immediately after UV exposure in the previous experiment, left open the possibility that the much higher percent survival rate of 9916 cells than that of E. coli cells after UV exposure was due to more effective LIR systems in 9916. Differentiating between these two types of repair pathways in E. coli is straightforward. Since heterotrophs such as E. coli grow as well in dark as in light, the contribution of photoreactivation pathways to cell survival after UV treatment is determined by measuring cell survival rate after UV treatment and subsequent WL exposure and then subtracting cell survival rate after UV treatment and subsequent dark exposure. But for photoautotrophs such as marine *Synechococcus*, dark exposure after UV treatment results in no cell growth, making it impossible to measure photoreactivation contributions to UV lesion repair and survival. We therefore searched for a light color other than blue light, which is the region of the spectrum known to photoactivate DNA photolyases, that would still allow 9916 cells to grow well. We found that 9916 cells grown under 10 μmol m^−2 ^ sec^−1^ of orange light (OL; wavelength maximum = 610 nm) grew slightly more than half as fast as cells grown in the same fluence rate of WL (Fig. S3).

10.1128/mbio.01511-22.3 FIG S3 Cell growth in orange light, white light, and dark. (A) *Synechococcus* sp. RS9916 cell cultures were split, plated, and grown for 21 to 23 days in OL (left) or 12 to 14 days in WL (right), and then CFU were counted. For no light (center), N.D. stands for no data, since these cells require light for growth. (B) E. coli cell cultures were split, plated, and grown in either OL (left), dark (center), or WL (right) and counted as described above. Error bars are the standard deviations of at least three independent replicates. Download FIG S3, PDF file, 0.8 MB. Copyright © 2022 Haney et al. 2022 Haney et al. https://creativecommons.org/licenses/by/4.0/ This content is distributed under the terms of the Creative Commons Attribution 4.0 International license.

Importantly, there was no apparent activation of the four Phr proteins by OL, since E. coli cells transformed with either *phr1*, *phr2*, *phr3*, or *phr4/5* driven by the pBAD24 arabinose-inducible promoter and exposed to either UV-B or UV-C irradiation all showed equivalent percent survival rates if the cells were subsequently either dark-treated or provided with 1 h of 10 μmol m^−2^ sec^−1^ of OL (Fig. S4), despite the fact that the proteins encoded by these genes are functional in E. coli cells (see below).

10.1128/mbio.01511-22.4 FIG S4 (A and B) Percent survival rates of *lexA3*Δ*phr* E. coli cells transformed with a plasmid either without or with the *Synechococcus* sp. RS9916 gene indicated on the *x* axis and treated with either (A) 153.6 J m^−2^ of UV-B or (B) 23.6 J m^−2^ of UV-C radiation. All treatments were followed either by 1 h of orange light and then continuous dark (orange bars) or by continuous dark (black bars). Survival rate is expressed as a percentage of cells counted on plates compared to the number of cells counted on plates that had not received any UV treatment. Error bars are the standard deviations of at least three independent replicates. Download FIG S4, PDF file, 0.8 MB. Copyright © 2022 Haney et al. 2022 Haney et al. https://creativecommons.org/licenses/by/4.0/ This content is distributed under the terms of the Creative Commons Attribution 4.0 International license.

**FIG 4:**
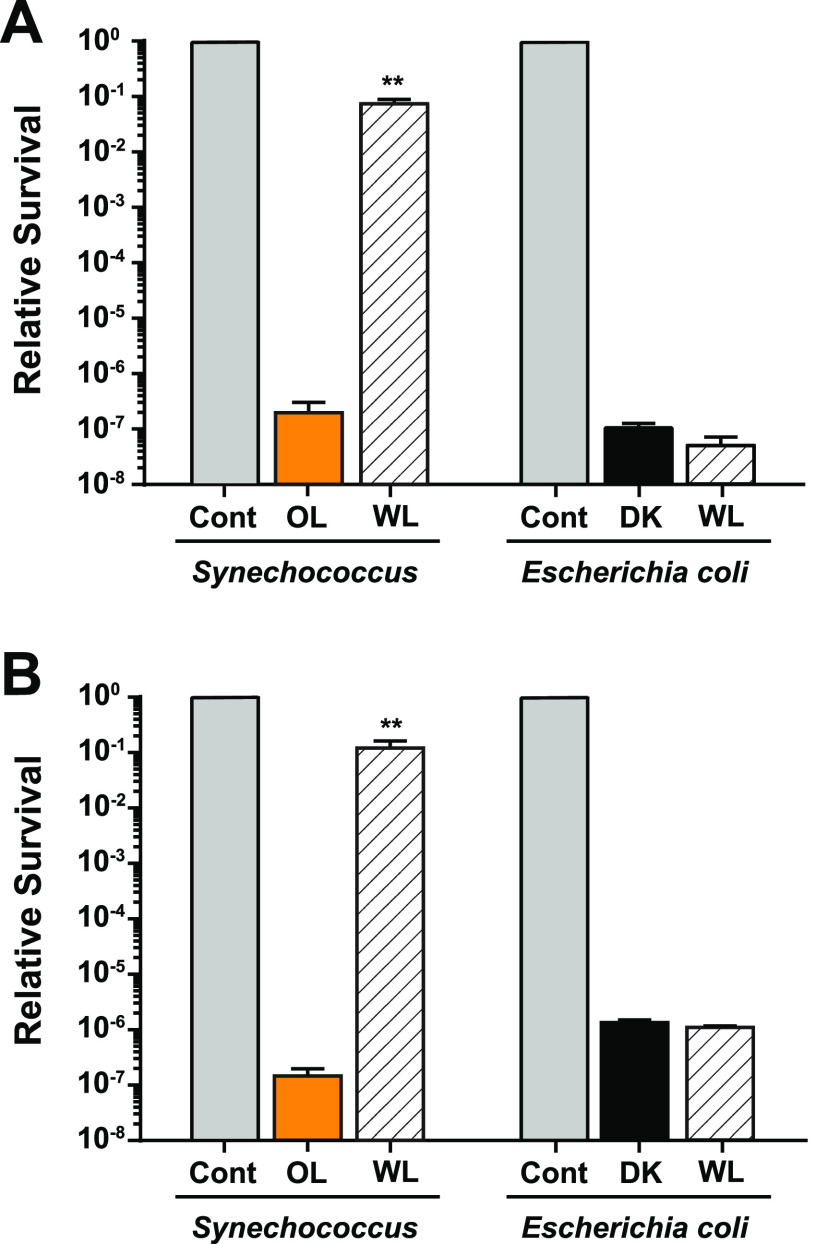
Light-dependent survival of *Synechococcus* 9916 and E. coli cells after UVR treatment. (A and B) After treatment with (A) 1,728 J m^−2^ of UV-B or (B) 212 J m^−2^ of UV-C, 9916 cells (left) were placed in either orange light (OL) or white light (WL), while E. coli cells (right) were placed in either the dark (DK) or white light (WL). After 1 h, a dilution series of cells were plated and grown under the same light conditions, and colony numbers were counted. For both organisms, a no-UV treatment control was included (Cont), and the values were normalized to 1. Error bars are the standard deviation (SD) of at least three replicates. **, *P* < 0.01.

The degree to which photoreactivation systems are used to repair UV-generated damage in marine *Synechococcus* was therefore assessed by using either a subsequent WL treatment to activate those systems or a subsequent OL treatment to simulate dark and not activate the systems. Parallel experiments were conducted for E. coli using either WL or dark as subsequent treatments. Separate UV-B and UV-C radiation treatments were carried out for both bacteria. The UV-B (Fig. 4A) and UV-C (Fig. 4B) doses given to these organisms decreased the percent survival rate of both by six to 8 orders of magnitude when followed by either OL or dark. However, when a WL treatment followed either UV-B or UV-C exposure, 9916 cell numbers only decreased by 1 order of magnitude compared to the no-UV controls, corresponding to a million-fold increase in percent survival rate relative to the OL-treated cells. In stark contrast, there was no statistically significant difference in the percent survival rate of wild-type E. coli cells that were subsequently provided a WL treatment versus a dark treatment. These data show that *Synechococcus* and E. coli have very different strategies for dealing with the damaging effects of both UV-B and UV-C, with photoreactivation processes playing a highly important role for *Synechococcus*, but LIR processes have greater importance for E. coli survival.

### Contribution of the putative photolyase genes to UVR survival in 9916.

We next examined the extent to which the proteins encoded by *phr1*, *phr2*, *phr3*, and *phr4* contributed to the WL-dependent increase in percent survival rate following UV-B and UV-C treatments in 9916. Four different interruption mutants were created using the pMUT100 suicide vector and verified by PCR amplification (Fig. S5). A previously constructed 9916 mutant containing pMUT100 (36, 37) was used as the “control cell” line that was grown with the same antibiotic selection as the interruption mutant. To determine whether this line and wild-type cells responded to UV treatment similarly, the effect of UV-B and UV-C radiation on the relative percent survival rate and growth of these control cells was compared to that of wild-type cells. The relative percent survival rate of the control cells was equivalent to that of wild-type cells after either UV-B or UV-C treatment and subsequent WL treatment, and the growth rates of these two cell types in WL, both prior to and after UV-C treatment, were also essentially the same (Fig. S6).

10.1128/mbio.01511-22.5 FIG S5 (A to D) Construction of four putative photolyase mutants in *Synechococcus* sp. RS9916. Diagrams showing the genomic regions of (A) *phr1*, (B) *phr2*, (C) *phr3*, and (D) *phr4* both prior to (above) and after (below) insertional mutagenesis. The size of the expected PCR amplification fragment for each interrupted gene, based on the primers listed in Table 1, is provided at the bottom of each diagram. (E) Ethidium bromide-stained agarose gel containing the PCR amplification products for each of the four pairs of primers used. Results of equivalent PCR amplification reactions using control cells (CC) are also shown for each primer pair. Molecular weight markers and sizes are shown on the left. Download FIG S5, PDF file, 1.3 MB. Copyright © 2022 Haney et al. 2022 Haney et al. https://creativecommons.org/licenses/by/4.0/ This content is distributed under the terms of the Creative Commons Attribution 4.0 International license.

10.1128/mbio.01511-22.6 FIG S6 Control cells grow and survive UV radiation similarly to wild-type cells. (A and B) Relative survival rates of *Synechococcus* sp. RS9916 wild-type cells (black bars) and control cells (gray bars) after treatment with (A) 212.4 J m^−2^ UV-C or (B) 1,728.0 J m^−2^ UV-B radiation. All cells were allowed at least 1 h of WL photoreactivation. (C and D) The growth of wild-type cells (black lines) and control cells (gray lines) in WL used for photoreactivation (C) without or (D) after a single dose of 141.6 J m^−2^ UV-C radiation, relative to the starting OD_750_. Error bars are the standard deviations of at least three independent replicates. Download FIG S6, PDF file, 0.1 MB. Copyright © 2022 Haney et al. 2022 Haney et al. https://creativecommons.org/licenses/by/4.0/ This content is distributed under the terms of the Creative Commons Attribution 4.0 International license.

**FIG 5:**
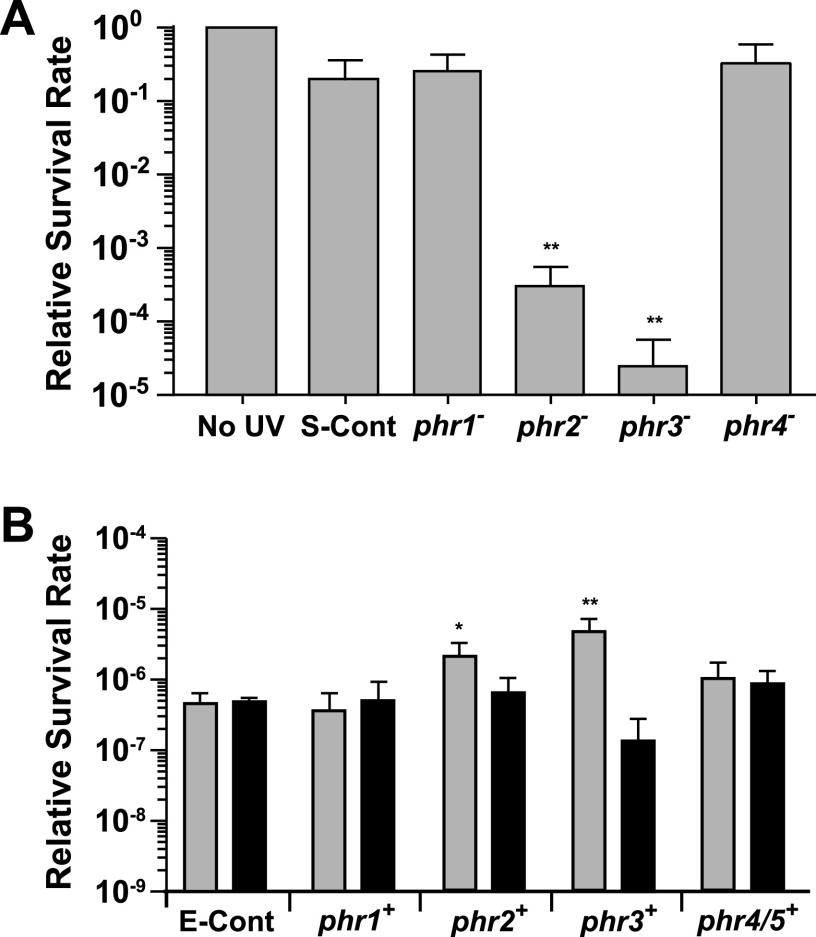
Contribution of putative photolyase-encoding genes to percent survival rates in *Synechococcus* and E. coli after UV-B treatment. (A) *Synechococcus* 9916 control cells were either not exposed to UV-B (No UV) or exposed to 1,728 J m^−2^ of UV-B followed by WL treatment (S-Cont), while four mutants containing insertions in putative photolyase-encoding genes (*phr1*, *phr2*, *phr3*, *phr4*) were given the equivalent UV-B and WL treatment. Values obtained for the no-UV control cells were set 1. **, *P* < 0.01, compared to control cells. (B) An E. coli mutant lacking photolyase activity and the SOS response was transformed with a vector only and either not exposed to UV-B or exposed to 154 J m^−2^ of UV-B (E-Cont) followed either by WL (gray bar) or dark (black bar) treatment. The same E. coli mutant was transformed with the same vector carrying either the *Synechococcus* 9916 *phr1*, *phr2*, *phr3*, or *phr4*/*phr5* gene and exposed to an equivalent dose of UV-B followed either by WL (gray bar) or dark (black bar) treatment. The data for the vector-only transformed *E. coli* cells that were not treated with UV-B were set to a value of 1 and are not shown in panel B. Error bars are the SD of at least three replicates. *, *P* < 0.05; **, *P* < 0.01, compared to light-treated control cells.

**FIG 6:**
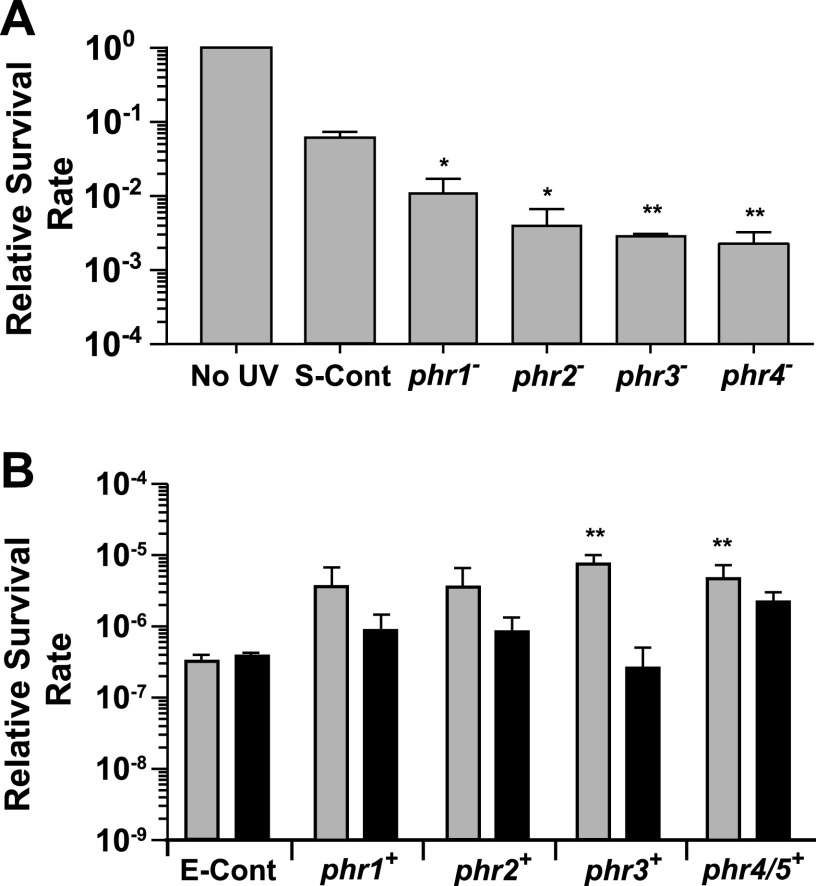
Contribution of putative photolyase-encoding genes to percent survival rates in *Synechococcus* and E. coli after UV-C treatment. (A) *Synechococcus* 9916 control cells were either not exposed to UV-C (No UV) or exposed to 212 J m^−2^ of UV-C followed by WL treatment (S-Cont), while four mutants containing insertions in putative photolyase-encoding genes (*phr1*, *phr2*, *phr3*, *phr4*) were given the equivalent UV-C and WL treatment. Values obtained for the no-UV control cells were set to a value of 1. **, *P* < 0.01, compared to control cells. (B) An E. coli mutant lacking photolyase activity and the SOS response was transformed with a vector only and either not exposed to UV-C or exposed to 24 J m^−2^ of UV-C (E-Cont) followed either by WL (gray bar) or dark (black bar) treatment. The same E. coli mutant was transformed with the same vector carrying either the *Synechococcus* 9916 *phr1*, *phr2*, *phr3*, or *phr4/5* gene and exposed to an equivalent dose of UV-C followed either by WL (gray bar) or dark (black bar) treatment. The data for the vector-only transformed *E. coli* cells that were not treated with UV-C were set to a value of 1 and are not shown in panel B. Error bars are the SD of at least three replicates. *, *P* < 0.05; **, *P* < 0.01, compared to light-treated control cells.

The growth rates of the *phr1* to *phr4* interruption mutants in WL were initially compared to those of both wild-type and control cells and found to be equivalent (Fig. S7). Each of the interruption mutants and the control cells were then exposed to either UV-B or UV-C radiation followed by WL, and the relative percent survival rate of each was compared to that of control cells that were not exposed to any UV treatment. Of the four insertion mutants, only the *phr2* and *phr3* disruptions led to decreased percent survival rate after exposure to the dose of UV-B radiation provided (Fig. 5A). The *phr2* mutant percent survival rate was approximately 1,000 times lower than that of control cells given the same amount of UV-B, and the *phr3* mutant percent survival rate was approximately 10,000 times lower than that of the UV-B-treated control cells. These results demonstrate that the *phr2* and *phr3* gene products strongly contribute to protection against UV-B damage in marine *Synechococcus*. Conversely, disruptions of neither *phr1* nor *phr4* had any measurable effect on the ability of these mutants to survive the exposure level of the UV-B used here, compared to the UV-B-treated control cells. When treated with UV-C radiation, all four mutants survived less well than the UV-C treated control cells, with the *phr1* mutant showing a 6 times lower percent survival rate and the *phr2*, *phr3*, and *phr4* mutants from 20 to 30 times lower percent survival rates (Fig. 6A). Taken together, the above-described results demonstrate that the products of each these four genes allow 9916 cells to better survive exposure to UV radiation.

10.1128/mbio.01511-22.7 FIG S7 *Synechococcus* sp. RS9916 control cells and *phr* mutant cells grow at the same rate in WL. Relative growth, as measured by absorbance at 750 nm (A_750_), of control cells (gray line), *phr1* mutant cells (green line), *phr2* mutant cells (purple line), *phr3* mutant cells (orange line), and *phr4* mutant cells (blue line). Error bars are the standard deviations of three independent replicates. Download FIG S7, PDF file, 0.1 MB. Copyright © 2022 Haney et al. 2022 Haney et al. https://creativecommons.org/licenses/by/4.0/ This content is distributed under the terms of the Creative Commons Attribution 4.0 International license.

### Contribution of the 9916 putative photolyase genes to UVR survival in E. coli.

A hallmark of photolyases is that they require light for their enzymatic activity. We investigated whether the protection from UV exposure provided to 9916 cells by the four putative photolyase genes was light dependent. We used an E. coli strain (Table 1) that lacked the endogenous *phr* gene as well as *lexA*, which disables the SOS response, making the cells more sensitive to DNA-damaging agents such as UVR and allowing any protection by the four 9916 photolyases to be more easily detected. This strain was transformed with either an empty vector or a vector containing one of the four *phr* genes from 9916 and exposed to either UV-B or UV-C radiation and then immediately given WL or placed in the dark. The percent survival rate of each transformed line was compared to that of E. coli cells transformed with the vector only, treated with UV light, and then exposed to either WL or dark. For transformants treated with UV-B, the percent survival rate of the lines carrying *phr2* and *phr3* was nearly 10 times higher than that of the vector-only control cells after a WL treatment but was the same as or lower than that of control cells after a dark treatment (Fig. 5B). This suggests that Phr2 and Phr3 9916 photolyases retain their activity when expressed in E. coli cells. No difference in percent survival rate was measured for transformants carrying *phr1* or *phr4* versus vector-only control cells with either a subsequent WL or dark treatment. Different results were obtained when these transformants were instead treated with UV-C (Fig. 6B). Compared to the control cells, all four transformants had much higher percent survival rates (10 to 20 times) when UV treatment was followed by a WL treatment, indicating that all four of these proteins provided the transformed E. coli cells with added protection from UV-C radiation. In addition, for the lines transformed with *phr1*, *phr2*, and *phr3*, the increased protection was lessened by 5 to 20 times when no WL was provided after UV-C treatment, demonstrating that light was essential for the high survival rates measured for these lines. For E. coli transformed with the DNA region containing *phr4* and *phr5*, the increased percent survival rate of cells measured after UV-C treatment was not significantly different with a subsequent WL versus dark treatment.

## DISCUSSION

Comparison of the genomes of 81 strains of the genera *Synechococcus*, *Cyanobium*, and *Prochlorococcus*, isolated from a variety of marine and brackish environments, showed that collectively, they possess eight different members of the photolyase/cryptochrome protein family. Among these, three complete *phr* genes (*phr1* to -*3*) were found in almost all picocyanobacterial strains examined in this study and encode two typical photolyases (*phr1* and *phr3*), consisting of a C-terminal FAD domain and an N-terminal photolyase domain, and one with a divergent N-terminal photolyase domain (*phr2*). Orthologs of *phr1* and *phr3* have been characterized in a number of model (cyano)bacteria. The *phr1*-like gene was found to encode an MTHF-type cryptochrome in V. cholerae (55), while the *phr3*-like gene encodes an MTHF-type CPD photolyase in V. cholerae (55) and A. tumefaciens (56) or an 8-HDF-type CPD photolyase in the cyanobacterium *Synechocystis* sp. strain PCC 6803 (6803) (57–59) and Synechococcus elongatus PCC 7942 (60, 61). In contrast, thus far, Phr2 has been identified and characterized as a 6,7-DMRL-type (6-4) photolyase only in V. cholerae and A. tumefaciens (43, 45, 46, 62), even though it appears to be present in all model cyanobacteria used in this study except the thylakoid-lacking Gloeobacter violaceus, which instead appears to possess a plant-like (6-4) photolyase (see Table S2) (63–65). Much of the variability between marine and brackish picocyanobacterial strains apparently relies on a fourth member of the photolyase/cryptochrome family, which can be one of three additional members of this family: first, *phr8* in Cyanobium gracile PCC 6307, which is orthologous to 6803 *phrB* (sll1629), once suspected to be a cryptochrome (66) and more recently characterized as a single-strand DNA CPD photolyase (67); second, a novel photolyase form with inverted FAD and photolyase domains, which is encoded either by two separated genes, *phr4* and *phr5*, in all marine *Synechococcus* and a number of *Cyanobium* strains or by a single gene, *phr6*, in two other *Cyanobium* strains; and third, a recently discovered single-strand DNA photolyase member, *phr7*, which consists of an N-terminal FAD domain and an atypical, short photolyase domain called the Z-domain (48) in all *Prochlorococcus* HL and LLI strains. It is also noteworthy that Nostoc punctiforme, although not closely phylogenetically related and living in a very different habitat (in symbiosis with plants) (68), has exactly the same *phr* gene complement as all marine *Synechococcus* isolates examined in this study. In addition, V. cholerae has a similar *phr* gene complement as *C. gracile* PCC 6307 except that its Phr2-like photolyase lacks the conserved cysteine residues required to bind an Fe-S cluster (see Table S2). More generally, all members of *Synechococcus* subcluster 5.1 and three out of six *Cyanobium* strains appear to contain forms of Phr2 that lack this Fe-S cluster and thus differ from characterized Phr2-like photolyases that have been shown to act as (6-4) photolyases (45, 46, 62). This suggests that the Phr2-like form found in most picocyanobacteria is likely to act as a CPD photolyase. In contrast, the novel photolyase sequences with reversed domain orders identified in this study, Phr4/Phr5 and Phr6, all possess the cysteines required to bind an Fe-S cluster, suggesting that they act as (6-4) photolyases, even though they are most closely related to Phr7, which is predicted to lack an Fe-S cluster (43) (Fig. 2). It is also interesting that although *Prochlorococcus* HL and LLI strains possess four members of the photolyase/cryptochrome gene family, none appear to encode a (6-4) photolyase, as is the case for Trichodesmium erythraeum (Table S2).

The arrangement of *phr4* and *phr5* in all marine *Synechococcus* and many *Cyanobium* strains (Fig. 1B) may lead to the formation of two single-subunit proteins that must heterodimerize to function correctly. However, it is curious that the spacing and sequence between these genes is so precisely maintained between different isolates. The presence of these genes as a single gene, *phr6*, in two *Cyanobium* strains suggests that the switching of the positions of the two domains may still create a functional photolyase. It also raises the possibility that in isolates with *phr4* and *phr5*, recoding may occur through a +1 programmed frameshift at or before the translation stop codon of *phr4* and lead to a final protein product that is structurally similar to Phr6. Such frameshifts are well known to occur in bacteria (44, 69). One example is a +1 frameshift in the *pfrB* gene, which encodes peptide release factor 2. This frameshift appears to be widespread and may occur in the cyanobacterium 6803 (70). If programmed frameshifting does occur between *phr4* and *phr5*, when it happens and the reason for it are not clear.

UV-C has been found to induce a greater ratio of 6-4:CPD lesions than UV-B (71). Our results are largely consistent with this finding. The loss of Phr4/Phr5, which we predict is a (6-4) photolyase, did not reduce survival after UV-B treatment (Fig. 5A), which should create a relatively low ratio of 6-4:CPD lesions. Additionally, the absence of either Phr2 or Phr3, which are both predicted to be CPD photolyases, significantly decreased cell survival after UV-B treatment (Fig. 5A). The converse was observed after UV-C treatment, which generates a higher ratio of 6-4:CPD lesions: loss of the possible (6-4) photolyase Phr4/Phr5 led to lower survival rates than did the loss of the putative CPD photolyases Phr1, Phr2, or Phr3 (Fig. 6A). The Phr1 results are not consistent with these findings, since it is proposed to be a CPD photolyase but provides less protection against UV-B than UV-C (Fig. 5A and 6A). Introduction of these 9916 genes into E. coli also mirrored these results to some extent. The presence of *phr4*/*phr5*, predicted to encode a (6-4) photolyase, had very little effect on the survival rate of E. coli cells after UV-B treatment. However, it did provide protection from UV-C, as expected (Fig. 5B and 6B). Phr2 and Phr3, which provided protection from UV-B in keeping with their proposed roles as CPD photolyases (Fig. 5B), also effectively protected E. coli cells from UV-C (Fig. 6B). And despite the predicted role of Phr1 as a CPD photolyase, the introduction of *phr1* in E. coli cells increased the relative survival rate after UV-C, but not UV-B, treatment (Fig. 5B and 6B). *In vitro* experiments will be required to determine the exact biochemical functions of these photolyases in 9916.

Even if *phr4*/*phr5* encodes a (6-4) photolyase, and the remaining three genes encode CPD photolyases, their conservation in all marine *Synechococcus* examined suggests that each of these is likely to have a unique role. One possibility is that their photoactivation wavelength optima differ. Because these cells experience a range of light colors in the environment, it may be advantageous to produce several photolyases with the same DNA repair function but different antenna chromophores, each maximally absorbing a different light color. For example, in coastal and estuarine environments, where blue light is less abundant in the water column, UV-generated DNA damage could be repaired by a photolyase family member that uses a color in the visible spectrum other than blue for photoactivation of the DNA repair process.

These enzymes are also likely to be produced in proportion to the amount of DNA damage the cell is experiencing. It has already been shown that these genes are differentially expressed after UVR exposure and during the diurnal cycle. Global transcriptomic analyses of the marine *Synechococcus* strain WH 7803 in response to changes in different environmental conditions has shown that there are differences in the expression of the five *phr* genes (6), suggesting that their products provide distinct benefits under various physiological states. Interestingly, this work found that transcript levels for *phr1*, *phr3*, *phr4*, and *phr5* in low-light-grown cells increased dramatically up to 6 h after UV-A and UV-B exposure, while *phr2* transcript levels did not. Because we found that the loss of *phr2* in 9916 led to significant decreases in survival after both UV-B and UV-C radiation, Phr2 may be a central, invariant component of the basal DNA repair response in these organisms. This is supported by the expression of these genes throughout the diurnal cycle in WH7803, with *phr2* transcripts being equally abundant throughout the day, possibly allowing Phr2 to provide continuous UV protection throughout the day, while *phr3*, *phr4*, and *phr5* transcript abundance levels were highest at 6 h after subjective dawn, when irradiance levels were at their peak, and then declined until subjective dusk, suggesting that the corresponding proteins provide the additional protection from UV damage when it was at its greatest (6). Analysis of the biochemistry of these proteins, as well as their expression levels with and without UVR exposure and throughout the light-dark cycle, would help to clarify each family member’s role in UVR protection.

9916 cells were able to survive much higher doses of both UV-B and UV-C than were E. coli cells (Fig. 3A and B). Unlike for E. coli, photoreactivation clearly plays a large role in the recovery of 9916 cells from both UV-B and UV-C exposure (Fig. 4A and B). The first photoreactivation of UVR-damaged DNA in a cyanobacterium was demonstrated over 50 years ago (72), and LIR has also been demonstrated in multiple species (73–75). While LIR has been found to lead to only partial recovery in cyanobacteria (73, 75), photoreactivation is essentially responsible for 100% of the survival in this group (59, 74, 76–79). Thus, photoreactivation seems to be a major repair pathway, not only for oceanic picocyanobacteria, as we have determined here (Fig. 4A and B), but also for cyanobacteria in general.

We were interested in comparing the 9916 percent survival rate after UVR treatment to that of other cyanobacterial species after similar treatments. In this study, we used relatively large doses of UVR for short time periods. Many investigations of both marine (2, 9, 10, 80–84) and freshwater (85–99) cyanobacteria have used lower UVR doses over longer time periods and examined phenotypes other than percent survival rate, making it difficult to compare to our results. In contrast, other studies have examined percent survival rates after high UV-C-only treatments. However, UV-B is the most damaging form of UVR in the natural environment since UV-C does not penetrate the Earth’s atmosphere, although over geologic time, superflares from the sun have periodically led to levels of all forms of UVR that have been far higher than Earth is currently experiencing (100). For these reasons we carried out two sets of studies. We used UV-C in order to compare our results to previous work and examine the response of marine *Synechococcus* to harsher UVR conditions. In addition, we used UV-B to gain a better understanding of the response of these organisms to large amounts of a form of UVR that they currently experience in nature.

Thus far, the only marine cyanobacterial strain examined for its percent survival rate after large doses of UVR is *Synechococcus* sp. strain PCC 7002 (7002), which was isolated from sediments below a fish pen in Puerto Rico (76). This study showed that after treatment with 90 J m^−2^ of UV-C and photoreactivation, 7002’s percent survival rate dropped by 1,000 times, whereas the same treatment for 9916 only led to a 3- to 5-fold decrease in the percent survival rate (Fig. 3B). Thus, it appears that 9916 is much more tolerant of UV-C than is 7002, even though the latter strain has been reported to tolerate up to 4,000 μmol photons m^−2^ sec^−1^ of white light, which corresponds to twice the maximum of full sunlight (101).

A number of freshwater cyanobacterial strains also have been tested for their UVR resistance and the contribution of photoreactivation to this process. The unicellular strains *Synechococcus* sp. strain PCC 6308 (6308) and *Synechocystis* sp. PCC 6803 (6803) were slightly more tolerant than 9916. Exposure of 6803 to 200 J m^−2^ of UV-C did not affect its percent survival rate (73) but decreased the 9916 percent survival rate by nearly 10-fold (Fig. 3B). The contribution of photoreactivation to overall percent survival rate after treatment with 200 J m^−2^ of UV-C appeared to be slightly greater for 9916, increasing the percent survival rate by a million-fold (Fig. 4B), but only by 100,000-fold for 6308. Even a milder treatment with 120 J m^−2^ of UV-C and subsequent photoreactivation, which did not affect 6803 survival (59), led to a 50% decrease in survival rate for 9916 (Fig. 3B). The freshwater unicellular strain Gloeocapsa alpicola was much more tolerant of UV-C exposure than was 9916. For *G. alpicola*, 600 J m^−2^ of UV-C with photoreactivating light resulted in only a 10-fold decrease in percent survival rate (79), while for 9916 there was a 10,000-fold decrease in percent survival rate after being treated with 650 J m^−2^ of UV-C and photoreactivating light (Fig. 3B). The 1,000-fold difference in UV-C percent survival rate between these two cyanobacteria may be partly explained by the thick exopolysaccharide coat of *G. alpicola*, which absorbs UVR well (102) and is absent in 9916. This was supported by the relatively small amount of photoreactivation for *G. alpicola*, whose percent survival rate after treatment with approximately 200 J m^−2^ of UV-C only increased approximately 100-fold, while the increase for 9916 was a million-fold (Fig. 4B). Two strains of the freshwater filamentous genus *Anabaena* also survived high doses of UV-C much better than 9916. For these strains, 700 J m^−2^ of UV-C radiation led to only a 10-fold drop in the percent survival rate (103), whereas for 9916, a comparable treatment caused a 10,000-fold decrease in percent survival rate (Fig. 3B). However, photoreactivation was much less important for *Anabaena* survival than for 9916, since after treatment with a UV-C dose of 200 J m^−2^, it only increased the percent survival rate 5-fold for *Anabaena* compared to a million-fold for 9916 cells (Fig. 4B). Only the unicellular, freshwater cyanobacterium *Synechococcus* sp. strain PCC 7942 has been found to be less UV-C tolerant than 9916. When treated with 90 J m^2^ and photoreactivating light, its percent survival rate decreased by approximately 10-fold (75), compared to a 5-fold decrease for 9916 (Fig. 3B).

Taken together, these results demonstrate that cyanobacteria vary widely in their UVR tolerance and that, after UVR exposure, photoreactivation processes are more important for the survival of marine *Synechococcus* than for most other cyanobacterial species. Our results provide the first molecular genetic analysis of how marine picocyanobacteria cope with high UVR levels in their natural environment and demonstrate the dramatic differences in the mechanisms through which photoautotrophic and heterotrophic bacteria deal with UVR. Another major finding of this study is the elucidation of the complete set of photolyase/cryptochrome family members present in a number of model heterotrophic bacteria and cyanobacteria. Of special note, we uncovered a novel photolyase family member with a new domain order, encoded either by *phr4-5* or *phr6*, that may act as a (6-4) photolyase and appears to be capable of operating either as a single, multidomain polypeptide or as a multisubunit enzyme. In the future, it will be interesting to determine how the activity of this family member differs from photolyases with a conventional domain order.

## MATERIALS AND METHODS

### Comparative genomics and phylogenetic analyses.

Most picocyanobacterial sequences were retrieved from the Cyanorak v2.1 database (http://www.sb-roscoff.fr/cyanorak; cf. accession numbers in Table S1), while the outgroup sequences, including the freshwater cyanobacteria *Synechocystis* sp. PCC 6803, Synechococcus elongatus PCC 7942, *Gloeobacter violaceus* PCC 7421, and Nostoc punctiforme PCC 73102, the marine cyanobacterium Trichodesmium erythraeum, and the heterotrophic bacteria V. cholerae O395 and E. coli K-12 were retrieved from GenBank (Table S2). A phylogenetic tree was generated from an alignment, made with MAFFT (104), of the FAD-binding domain of Phr1, 2, 3, 6, 7, and 8 and the whole sequences of Phr4, the photolyase domain being too variable to achieve reliable alignments of entire protein sequences. Phylogenetic reconstructions were performed using maximum likelihood (ML; PhyML v3.3), neighbor-joining (NJ; Phylip 3.69), and maximum parsimony (MP; Phylip 3.69) using 100 bootstrap replicates. ML reconstructions were performed using the Le and Gascuel substitution model, as determined using ProtTest v3.4.1 (105). All reconstructions were visualized using Archaeopteryx v0.9901 (106), and the tree was drawn using iTOL (107).

### RS9916 strains and growth conditions.

Control cells and growth conditions were similar to those previously described (36). Cultures were grown semicontinuously in polycarbonate culture flasks at 22°C in PCR-S11 medium under constant irradiance of either 10 μmol photons m^−2^ s^−1^ WL (Chroma 75 T12; General Electric) or OL (custom-built light-emitting diode panels; Digi-Key part no. 754-1084-2-ND). A list of strains used for experimental work is provided in Table 1.

### E. coli strains and growth conditions.

E. coli strains (Table 1) were grown at 37°C in LB medium with or without 100 μg/mL ampicillin and shaken at 150 rpm. Cultures with plasmids were induced with 10 mM (approximately 0.15%) l-arabinose and grown to an optical density at 600 nm (OD_600_) of 0.6 to 0.8. When grown in WL, Solux 4,700 K halogen lamps were used (Eiko Ltd.; catalog no. Q50MR16/CG/47/36). A list of strains and plasmids used for experimental work is provided in Table 1.

### Construction of RS9916 strains.

The plasmids and primers used are listed in Table 1. Mutant plasmids were made via PCR amplification of an internal ca. 500-bp region of each gene cloned into pMUT100. Insertion mutations of the genes were generated by conjugation as previously described (36). Individual colonies were selected and screened via PCR amplification for proper insertions. PCR-amplified DNA was sequenced to verify the insertion. Cultures were maintained with 50 μg μL^−1^ kanamycin when grown in liquid medium.

### Construction of E. coli strains.

E. coli strains were constructed in a *lexA3*Δ*phr* background (lacks the SOS response). The photolyase genes were PCR-amplified from RS9916 genomic DNA and cloned into the pBAD24 plasmid under the control of the arabinose-inducible promoter. Individual colonies were selected and screened via PCR amplification for proper insertions. DNA was sequenced to verify the insertion. Cultures were maintained with 100 μg μL^−1^ ampicillin when grown in liquid medium.

### RS9916 UV treatments.

Cells were diluted to mid-log phase based on the OD_750_ and exposed to differing amounts of either UV-C (Sylvania germicidal primarily emitting at 254 nm, SG818-D) or UV-B light (Ushio primarily emitting at 306 nm, G8T5E) as a thin liquid layer just covering the bottoms of 60 by 15-mm petri dishes. Cells were then immediately treated for 1 h with either 10 μmol photons m^−2^ s^−1^ WL (Chroma 75 T12; General Electric) or OL (custom-built light-emitting diode panels; Digi-Key part no. 754-1084-2-ND) and then plated in 0.3% agar plates and maintained under the same light conditions. Colonies were counted on the plates between 12 and 16 days after treatment. The UV dose was monitored using a UVX radiometer (UVP, Inc., San Gabriel, CA) equipped with a UVX-25 sensor for 254-nm UV-C radiation and a UVX-31 sensor for 306-nm UV-B radiation.

### E. coli UV treatments.

Cells were diluted to mid-log phase, based on the OD_600_ and then plated on 1% LB agar plates and exposed to different amounts of either UV-C (Sylvania germicidal primarily emitting at 254 nm, SG818-D) or UV-B light (Ushio primarily emitting at 306 nm, G8T5E). Cells were then immediately moved to either WL or dark for 1 h and then incubated at 37°C overnight. The UV dose was monitored using a UVX radiometer equipped with a UVX-25 sensor for 254-nm UV-C radiation and a UVX-31 sensor for 306-nm UV-B radiation.
