# Supplementary figures and images for: Multiple Photolyases Protect the Marine Cyanobacterium Synechococcus from Ultraviolet Radiation
Source: mBio. 2022 Jul 20;13(4):e01511-22. doi: 10.1128/mbio.01511-22 (PMC9426592; doi:10.1128/mbio.01511-22)

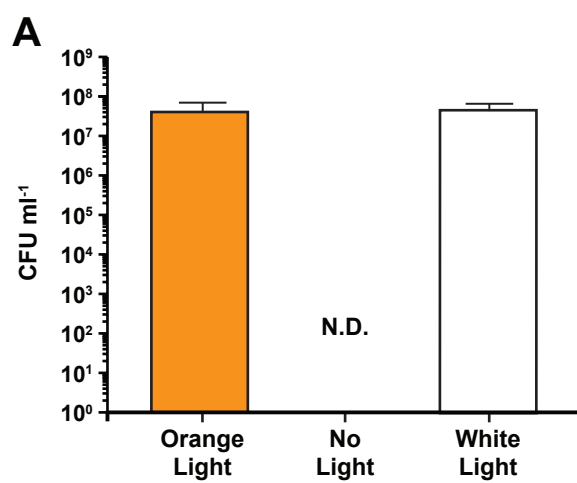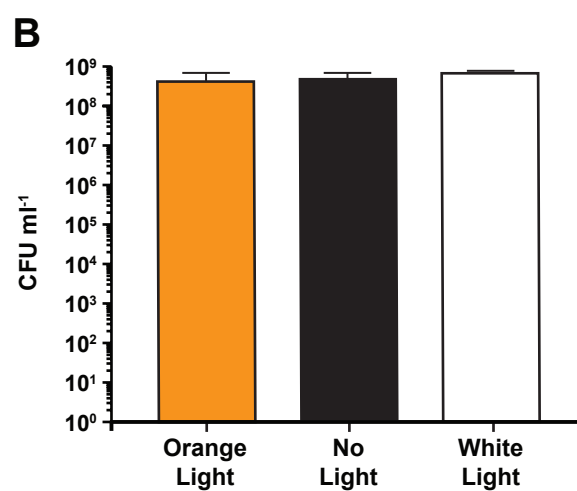

Supplement: FIG S3 [file mbio.01511-22-s0003.pdf]

**A**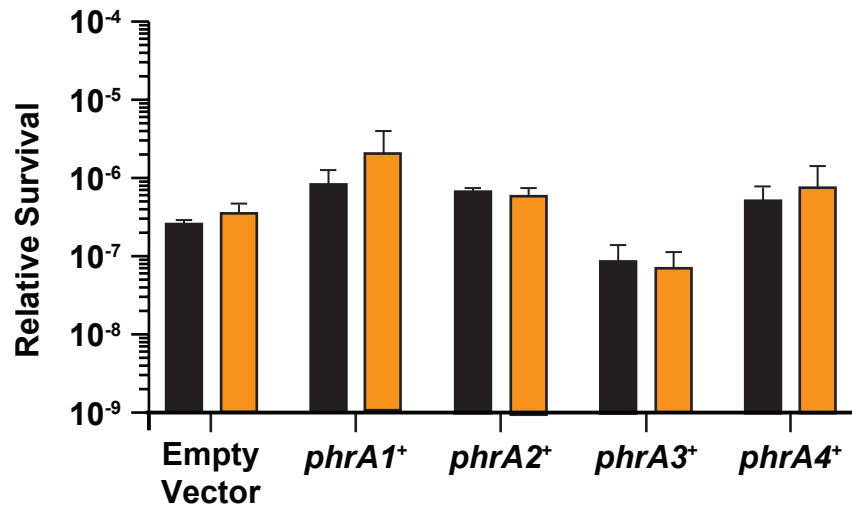**B**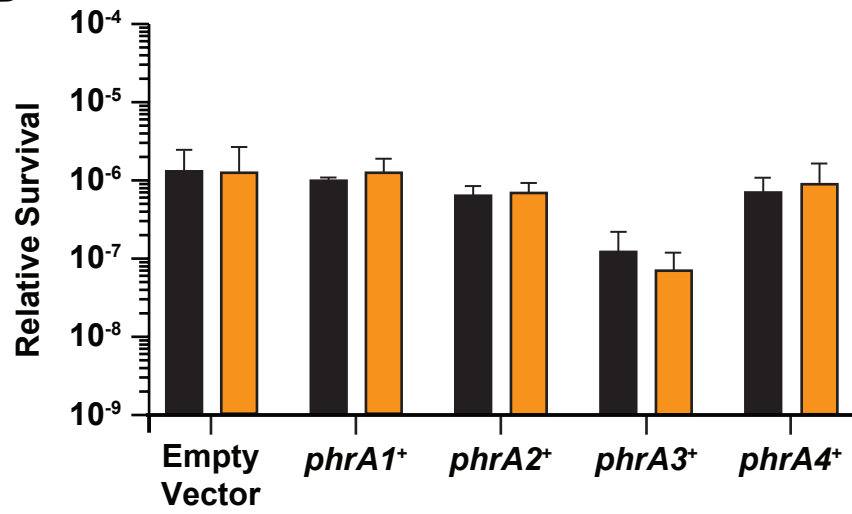

Supplement: FIG S4 [file mbio.01511-22-s0004.pdf]

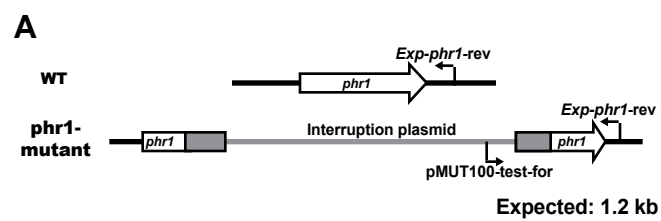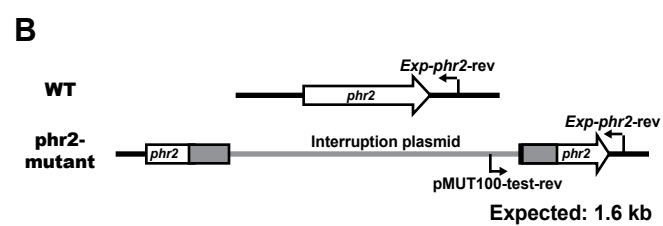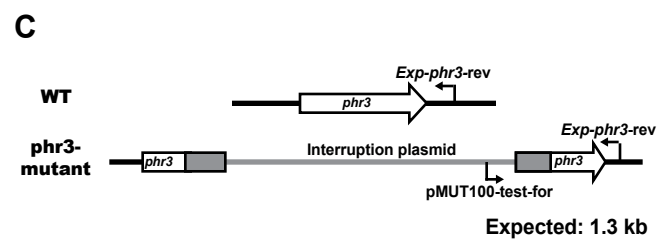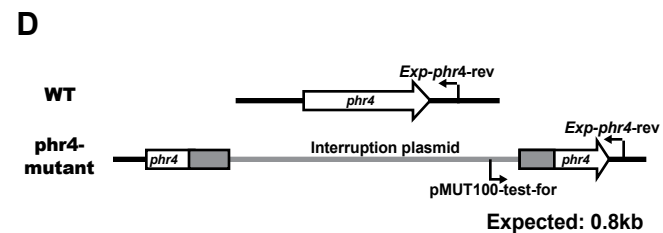

**E**

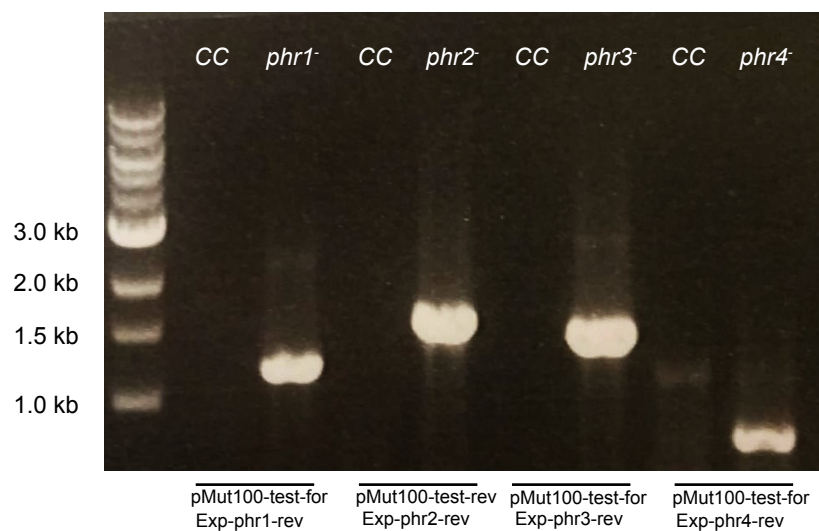

Supplement: FIG S5 [file mbio.01511-22-s0005.pdf]

**A**

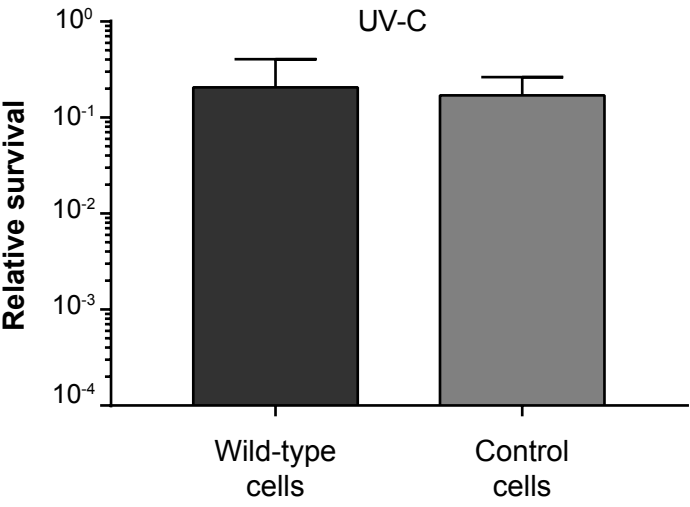

**B**

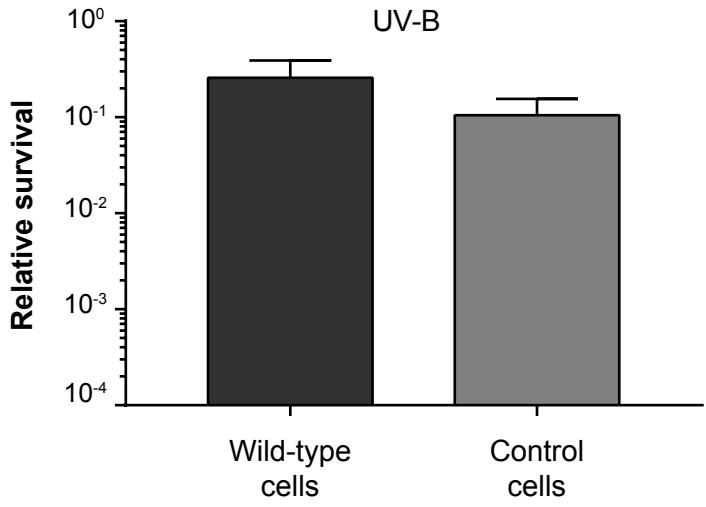

**C**

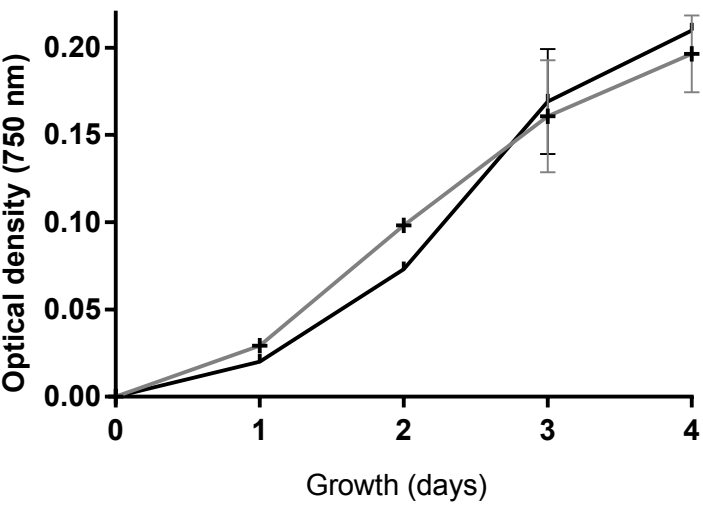

**D**

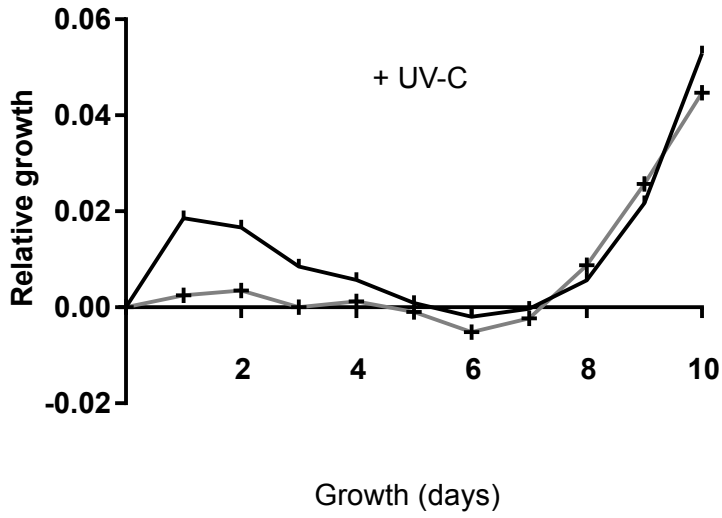

Supplement: FIG S6 [file mbio.01511-22-s0006.pdf]

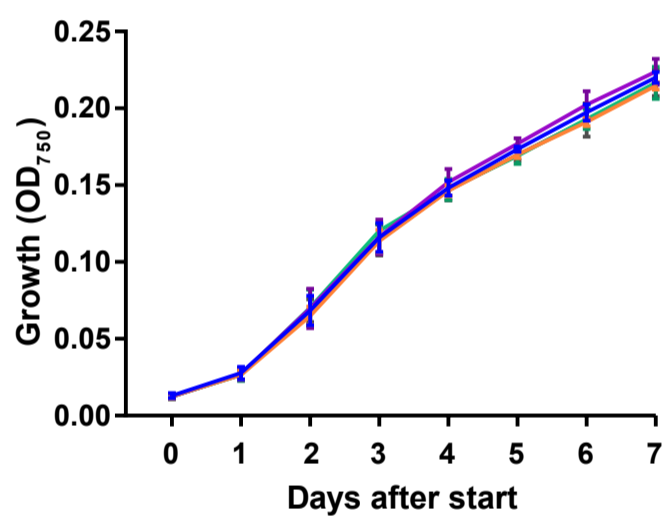

Supplement: FIG S7 [file mbio.01511-22-s0007.pdf]
